# Single-Image Visibility Restoration: A Machine Learning Approach and Its 4K-Capable Hardware Accelerator

**DOI:** 10.3390/s20205795

**Published:** 2020-10-13

**Authors:** Dat Ngo, Seungmin Lee, Gi-Dong Lee, Bongsoon Kang

**Affiliations:** Department of Electronics Engineering, Dong-A University, Busan 49315, Korea; datngo@donga.ac.kr (D.N.); 1672885@donga.ac.kr (S.L.); gdlee@dau.ac.kr (G.-D.L.)

**Keywords:** haze removal, machine learning, supervised learning, hardware accelerator, field programmable gate array

## Abstract

In recent years, machine vision algorithms have played an influential role as core technologies in several practical applications, such as surveillance, autonomous driving, and object recognition/localization. However, as almost all such algorithms are applicable to clear weather conditions, their performance is severely affected by any atmospheric turbidity. Several image visibility restoration algorithms have been proposed to address this issue, and they have proven to be a highly efficient solution. This paper proposes a novel method to recover clear images from degraded ones. To this end, the proposed algorithm uses a supervised machine learning-based technique to estimate the pixel-wise extinction coefficients of the transmission medium and a novel compensation scheme to rectify the post-dehazing false enlargement of white objects. Also, a corresponding hardware accelerator implemented on a Field Programmable Gate Array chip is in order for facilitating real-time processing, a critical requirement of practical camera-based systems. Experimental results on both synthetic and real image datasets verified the proposed method’s superiority over existing benchmark approaches. Furthermore, the hardware synthesis results revealed that the accelerator exhibits a processing rate of nearly 271.67 Mpixel/s, enabling it to process 4K videos at 30.7 frames per second in real time.

## 1. Introduction

The world is currently going through the fourth industrial revolution (also known as 4IR or Industry 4.0) and is ’on the cusp’ of the fifth one (5IR or Industry 5.0). In particular, machine vision algorithms play an influential role in 4IR and 5IR technologies due to their rapid development over the last few decades. They have appeared in various systems, including autonomous driving vehicles, driver-assistance systems, and smart surveillance cameras. However, weather conditions and atmospheric turbidities such as haze, snow, and yellow dust have affected such systems’ accuracy adversely, threatening operational failures that could lead to unfortunate consequences. For example, adverse weather conditions severely affect the maritime surveillance systems (e.g., ship tracking [1]), whose accuracy and performance consistency are of great importance. Thus, various algorithms have come into use to address scene visibility degradation, and they primarily fall into two categories: multi- and single-image techniques. While those belonging to the former category usually outperform those belonging to the latter in terms of the quality of image enhancement, their requirement of extensive external knowledge engenders multiple practical difficulties. Therefore, the latter type algorithms garnered increasing interest among researchers over the past decade, and researchers have approached them from two perspectives—image enhancement and image restoration.

Histogram equalization [2,3], low-light stretching [4], unsharp masking [5,6], and homomorphic filtering [7,8] are fast-and-straightforward image enhancement techniques. They are highly efficient when the captured scene is slightly hazy because they primarily enhance low-level features such as edges, brightness, and contrast, which significantly influence human perception of image quality. Nevertheless, as these methods do not take the cause of distortion into account, the effects of atmospheric turbidity persist, inducing unsatisfactory visual perception. An example of such a method is the nonlinear unsharp masking algorithm presented in [5]. This method begins by decomposing each input image into constituent background and detail signals, followed by enhancement of the detail signal via an adaptive gain and optional contrast enhancement of the background signal. Finally, the sum of two signals takes place to obtain the output image with enhanced contrast and sharpness. It is worth noticing that all these operations are executed by generalized operators to avoid the out-of-range problem. A qualitative evaluation of the aforementioned algorithm reveals that, while faded details of hazy images were of significant enhancement, the haze persisted in the enhanced images.

On the other hand, image restoration techniques for single-image visibility enhancement have improved upon the aforementioned methods by taking the cause of image distortion into account. In this context, Koschmieder’s law [9], which describes the multiplicative attenuation of scene radiance and additive light scattering, has been used to model image distortion. While Section 2.1 will explain Koschmieder’s law in greater detail, its ill-posed nature merits attention. The impossibility of direct recovery of clear visibility from a sole input image gives rise to this ill-posed problem. Accordingly, strong priors or assumptions are essential to facilitate the restoration process. A series of studies in this direction [10,11,12,13,14] are prime examples. In these studies, prior knowledge about the image to be recovered—such as partial uncorrelatedness between the propagation of projected light and surface shading [10], attenuated saturation [13], and the distribution of color pixels in Red-Green-Blue (RGB) space [14]—was assumed to estimate the optimal values of the parameters appearing in Koschmieder’s law. Due to their dependence on such imposed priors, the aforementioned methods run the risk of failure under particular circumstances. It is worth noticing that visibility restoration algorithms from the image enhancement perspective recently exploited haze-relevant image priors [15] and multi-scale processing [16] to improve the restoration performance.

This paper proposes a single-image method to restore scene visibility based on Koschmieder’s law. As atmospheric scattering usually increases brightness and decreases saturation, it is efficient to use a prior proposed in [13] to estimate the atmosphere’s extinction coefficients via a machine learning-based method. Additionally, a parallel computing scheme inspired by the quad-decomposition algorithm proposed in [17] establishes a hardware-friendly means to estimate the atmospheric light. Furthermore, to overcome the drawbacks of current state-of-the-art methods, several ideas are discussed to remove background noise, color distortion, and the side-effect of false enlargement of white objects. Finally, to facilitate real-time processing, a hardware accelerator is designed with noticeable novelties to maximize the processing speed. The main contributions of this paper may be summarized as follows.

- This study is the first attempt to address the problem of the false enlargement of white objects. Based on the observation of failure of current methods in estimating atmospheric light in scenes containing white objects, an adaptive compensation scheme is proposed to offset the light in such cases.
- Prior to the aforementioned compensation step, a parallel algorithm is developed based on quad-decomposition to estimate the atmospheric light coarsely. This newly proposed method is beneficial to the hardware implementation phase due to eliminating burdensome image buffers and is a substantial contributor to the hardware architecture’s 4K capability.
- Furthermore, a novel hardware architecture was developed to realize the modified hybrid median filter. Although the previously developed architecture based on Batcher’s sorting network [18] is considerably compact and fast, the proposed design, which exploits both sorting and merging networks, is established to be even more efficient. This newly proposed architecture significantly contributes to the 4K capability of the proposed hardware accelerator.

The rest of this paper is organized as follows. The preliminary knowledge is introduced in Section 2, including Koschmieder’s law and typical visibility restoration algorithms. The proposed method is discussed in detail in Section 3 and the experimental validations are provided. The hardware accelerator is presented in Section 4 alongside the hardware synthesis results. Finally, the paper is concluded in Section 5.

## 2. Preliminaries

### 2.1. Koschmieder’s Law

Koschmieder’s law [9] describes the formation of images in turbid atmosphere conditions, and is as follows.

(1) $$E\left(x,\lambda \right)=e^{-\beta (\lambda )d(x)}E_{0}\left(x,\lambda \right)+\left[1-e^{-\beta (\lambda )d(x)}\right]E_{\infty }\left(\lambda \right),$$

where *E* and $E_{0}$ denote the scene radiance of the observed image and the clear image, respectively. $E_{\infty }$ denotes the observed scene’s lightness, *x* denotes the horizontal and vertical coordinates of pixels, λ denotes the wavelength of visible light, β denotes the extinction coefficient of the atmosphere, and *d* denotes the scene depth. The first term on the right-hand side denotes the direct attenuation, representing the multiplicative attenuation of the scene radiance in the transmission medium. The second term denotes the airlight, representing the additive scattering of the lightness. It is convenient to define I(x)=E(x,λ), $J\left(x\right)=E_{0}\left(x,\lambda \right)$, $t\left(x\right)=e^{-\beta (\lambda )d(x)}$, and $A=E_{\infty }\left(\lambda \right)$ for ease of expression, subsequently reducing Equation (1) to the following.

(2) $$I\left(x\right)=t\left(x\right)J\left(x\right)+\left[1-t(x)\right]A,$$

where *t* and A are now referred to as the transmission map and the atmospheric light, respectively. The symbols I, J, and A are written in bold as they possess three color components. According to the Rayleigh scattering phenomenon, the wavelength-dependent β(λ) induces t(x) to vary with respect to the color channels. However, this dependency is assumed to have a negligible impact on the accuracy of Equation (2) in almost all visibility restoration algorithms. As I(x) is the sole input captured by sensors, recovery of the clear scene radiance J(x) is an ill-posed problem due to the unknown variables t(x) and A. Therefore, the goal of visibility restoration is to estimate t(x) and A by imposing some priors on J(x) and subsequently obtain the clear scene radiance via the following equation.

(3) $$J\left(x\right)=\frac{I(x)-A}{t(x)}+A.$$

### 2.2. Related Work

In the literature, visibility restoration is also known as haze removal, dehazing, or defogging because atmospheric turbidity is universally referred to as haze or fog. Accordingly, in this paper, we have used these terms interchangeably. Recent studies on single-image visibility restoration generally fall into three main categories: simple image processing, machine learning, and deep learning-based techniques.

The dark channel prior (DCP) proposed by He et al. [11] is a prime example of a restoration technique belonging to the first category. Based on extensive observation of clear outdoor images, the authors discovered that most local non-sky patches contain some pixels that possess very low intensities in at least one color channel. Assuming this prior, they estimated the transmission map by using a channel-wise minimum operator followed by a local minimum filter. Additionally, soft matting [19] was adopted to refine the transmission map to suppress halo artifacts. Although DCP demonstrated good dehazing performance and broad applicability (e.g., underwater image restoration [20]), it is computationally expensive due to soft matting use. This drawback left room for improvement and thereafter inspired researchers to several approaches [21,22,23,24]. He et al. [25] also proposed a multi-function guided filter, which could replace soft matting to ease the burden of expensive computations at the cost of a certain degree of degradation in image quality. Gibson et al. [26] improved upon DCP and proposed the median dark channel prior, eliminating the step of transmission map refinement, thereby significantly accelerating the dehazing process. However, this elimination induced unsatisfactory enhancement quality. Kim et al. [27] presented a fast approach that employed a modified hybrid median filter to estimate the airlight. This filter, equipped with good edge-preserving characteristics, was used to exclude the refinement step, thereby accelerating the processing rate. However, post-dehazing background noise is the main drawback of this method [27].

Machine learning-based techniques such as maximum likelihood estimates (MLE), random forest regression, and support vector machine belong to the second category. They have been used by various researchers to restore clear visibility to images. Zhu et al. [13] identified a correlation between scene depth and the difference between an image’s saturation and brightness. Based on this observation, they proposed a linear model called color attenuation prior (CAP) to estimate the scene depth, which is exponentially proportional to the transmission map. CAP’s parameters were estimated using MLE under supervised learning, and a guided filter was used to refine the depth map. This method functions well in most circumstances except dark scenes, in which post-haze-removal background noise and color distortion are possible. Another machine learning-based algorithm proposed by Tang et al. [28] extracts haze-relevant features from an input image. It then transmits them to a random forest regressor to calculate the transmission map. Ngo et al. [29] proposed a similar method, exploiting the Nelder-Mead direct search algorithm to calculate the optimal transmission map. They also devised an adaptive atmospheric light to prevent the loss of dark details. Although the methods proposed by Tang et al. [28] and Ngo et al. [29] exhibit good dehazing performance, they are inappropriate for practical applications owing to their high time consumption. Choi et al. [30] proposed two approaches named fog aware density evaluator (FADE) and density of fog assessment-based defogger (DEFADE) for haze density assessment and haze removal, respectively. FADE computes the haze-relevant features from a collection of 500 hazy images and fits the features to a multivariate Gaussian model. It performs the same procedure on a collection of 500 haze-free images. The calculated mean vectors and covariance matrices establish the ground truth for haze density evaluation. DEFADE executes dehazing by using image fusion following the Laplacian pyramid scheme with corresponding weights calculated from haze-relevant features. However, DEFADE is also a computationally expensive method.

Finally, a recent research trend of applying deep learning-based methods to haze removal has also been observed. Cai et al. [31] proposed an end-to-end convolutional neural network (CNN), which accepts an input image and produces a corresponding transmission map. However, this method’s performance is limited, owing to the lack of real training datasets comprising pairs of hazy and haze-free images of the same scenes. Other studies presented in [32,33,34] have attempted to improve dehazing performance by increasing the receptive field via deeper CNNs or developing a sophisticated loss function instead of the widely employed mean squared error. However, the aforementioned lack of real training datasets continues to affect their results partially. Another shortcoming that might limit the broad deployment of deep learning-based approaches is their high computational cost. Currently, the graphics processing unit is the primary means for realizing such approaches, which has made the implementation of deep neural networks at end devices an active research area in recent years. Interested readers are referred to a comprehensive work conducted by Li et al. [35], which provides a thorough evaluation of traditional and deep learning-based haze removal methods.

**Figure 1:**
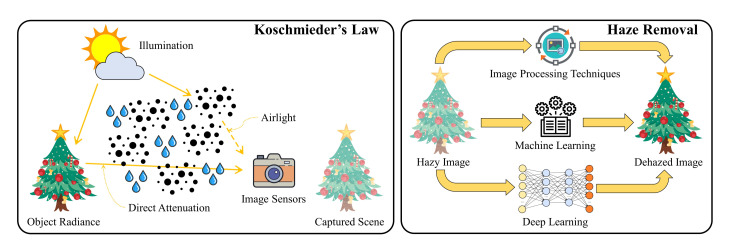
Visual illustrations of Koschmieder’s law and the three main categories of haze removal techniques.

Figure 1 summarizes this section by providing visual illustrations of Koschmieder’s law and the three main categories of haze removal techniques. The sun represents the light source whose emitting light waves traverse the turbid transmission medium represented by dust and water droplet icons. Accordingly, the captured image exhibits a faint color induced by direct attenuation and atmospheric scattering. Researchers developed various algorithms for restoring the image visibility in such a case, and the developed algorithms generally fall into three main categories. This paper named the categories according to their underlying technique, including image processing, machine learning, and deep learning.

## 3. Proposed Algorithm

This study is an extension of our previous work (i.e., Ngo et al. [36]) and can be characterized by three new improvements:

- a solution to the issue of false enlargement of white objects,
- an image buffer-free parallel computing scheme for atmospheric light estimation,
- and an optimized merging sorting network to implement the modified hybrid median filter.

**Figure 2:**
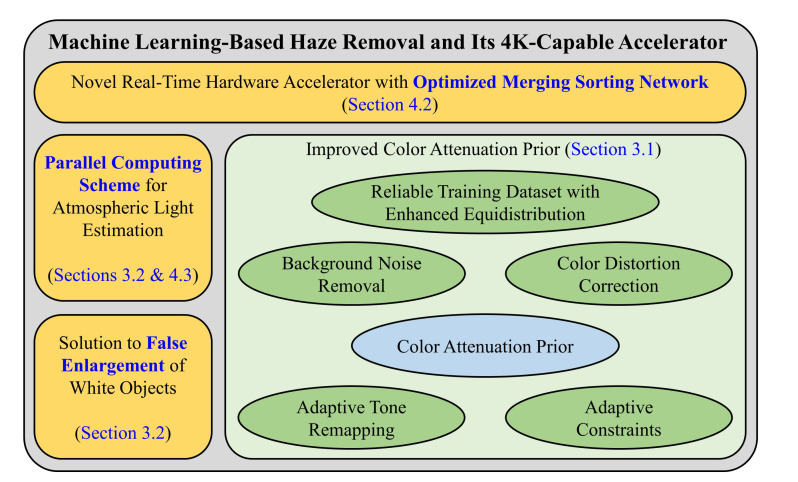
The proposed algorithm with its main contributions to the previous work.

Of the three points mentioned above, the first one is for the base algorithm, and it is seemingly a first attempt to deal with the false enlargement problem. The last two points are for the hardware counterpart, and they play an essential role in facilitating the real-time processing of 4K images/videos. Figure 2 depicts an overview of the proposed algorithm regarding its main contributions to the previous work. Our improved color attenuation prior (ICAP) [36] was developed based on the method of Zhu et al. [13] by adding several features such as enhanced equidistribution, adaptive constraints for the transmission map, background noise removal, color distortion correction, and adaptive tone remapping. The proposed algorithm completes the ICAP by integrating the three aforementioned characteristics. In the following subsections, the previous novelties of ICAP are first briefly presented to provide an adequate context for the subsequent discussion of the newly proposed ones.

### 3.1. Improved Color Attenuation Prior

#### 3.1.1. Enhanced Equidistribution for a More Reliable Training Dataset

**Figure 3:**
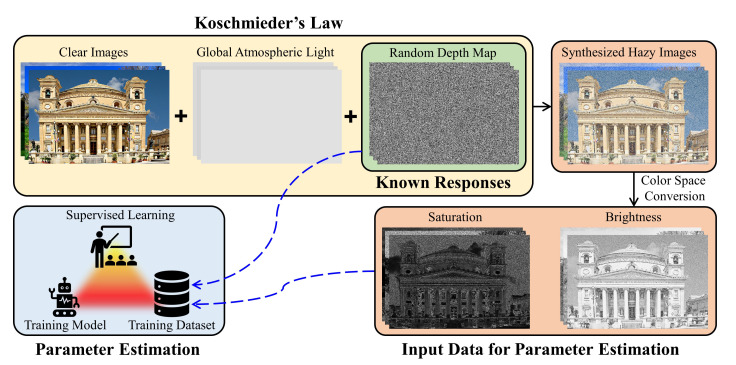
The procedure of preparing the synthetic training dataset for supervised learning-based parameter estimation.

The linear model proposed by Zhu et al. [13] for estimating the scene depth, d(x), based on the difference between an image’s saturation, s(x), and brightness, v(x), is as follows.

(4) $$d\left(x\right)=a_{0}+a_{1}s\left(x\right)+a_{2}v\left(x\right)+\varepsilon \left(x\right),$$

where $a_{0}$, $a_{1}$, and $a_{2}$ denote the model’s parameters, and ε(x) denotes the model’s error. For parameter estimation, collecting a training dataset consisting of hazy images and their corresponding scene depths is essential. However, this task appears to be infeasible due to the complete lack of reliable means to capture scene depth. Hence, Zhu et al. [13] proposed the three-step procedure illustrated in Figure 3 to prepare the training dataset. They first collected 500 clear images from image-sharing services such as Google Image, Flickr, and Pinterest. Then, corresponding to each image, random numbers drawn from the uniform distribution were used as the corresponding measurements of scene depth and atmospheric light. Finally, Koschmieder’s law was employed to synthesize the hazy images, whose saturation and brightness were included in the training dataset for parameter estimation in addition to the random depth maps.

**Figure 4:**
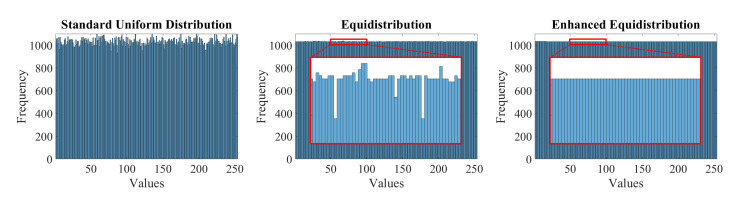
Histograms of data drawn from standard uniform distribution, equidistribution, and enhanced equidistribution.

Since current pseudo-random number generators do not guarantee the uniform distribution, the enhanced equidistribution developed in our previous work [37,38] is used as a surrogate for the standard uniform distribution to prepare the training dataset in this study. Figure 4 depicts three histograms of 262,144 random numbers drawn from the standard uniform distribution, the equidistribution [37], and the enhanced equidistribution [38], respectively. Although the leftmost set of values follows the uniform distribution, its standard deviation is relatively high. In contrast, the two right ones resemble the theoretical uniform distribution significantly, inducing better quantitative evaluation, as presented in [37,38]. The cropped regions highlighted in red further demonstrate the superiority of the enhanced equidistribution over the equidistribution as it resembles the theoretical uniform distribution more closely.

#### 3.1.2. Adaptive Constraints for The Transmission Map

The value of the transmission map presented in Equation (2) lies within the range (0,1] and is inversely proportional to the image’s haze density. Due to the existence of clear regions in most images, it is reasonable to retain the transmission map’s upper bound to be 1. Conversely, because image regions rarely become obscured by atmospheric turbidity entirely, Zhu et al. [13] limited the transmission map by instituting a fixed lower bound. In ICAP [36], by exploiting the linearity of Koschmieder’s law, two adaptive constraints for preventing the over-removal of haze were devised and then combined with the upper bound, as follows.

(5) $$max\left\{1-min_{c\in \{R,G,B\}}\left[\frac{I^{c}\left(x\right)}{A^{c}}\right],1-\frac{mean_{y\in \Omega (x)}\left[I^{gray}\left(y\right)\right]-f\cdot std_{y\in \Omega (x)}\left[I^{gray}\left(y\right)\right]}{\bar{A}}\right\}\leq t\left(x\right)\leq 1,$$

where *y* denotes the pixel location inside the square window Ω(x) centered at *x*, $\bar{A}$ denotes the channel-wise average of A, *f* denotes the user-defined parameter to control the tightness of the imposed constraint proportionally, mean(·) denotes the mean filter, and std(·) denotes the standard deviation filter.

#### 3.1.3. Solutions for Background Noises and Color Distortion

The algorithm proposed by Zhu et al. [13] suffers from background noise and color distortion, according to our previous investigation [36]. The cause of background noise was successfully traced back to spike-like noise in the saturation channel, and the linearity of Equations (3) and (4), which propagate the noise to the restored image. Hence, a simple low-pass filter with a normalized cut-off frequency of 0.16π radians/sample was applied to the saturation channel to suppress the noise. Concerning color distortion, dark regions with low saturation and brightness were discovered to be frequently misinterpreted as close regions by Equation (4). Thus, the uneven removal of haze is the fundamental cause underlying color distortion. The adaptive weight given by Equation (6) was proposed to ensure the execution of haze removal on dark regions as well.

(6) $$\omega_{t}\left(x\right)=\left(\frac{1-\omega_{0}}{d_{0}}\right)d\left(x\right)+\omega_{0},$$

where $\omega_{0}$ and $d_{0}$ denote user-defined parameters for specifying the gain in close regions and the close regions themselves. The equation for scene radiance recovery was revised as follows using the aforementioned weighting scheme.

(7) $$J\left(x\right)=\frac{I\left(x\right)-A\left[1-\omega_{t}\left(x\right)t\left(x\right)\right]}{t(x)}.$$

#### 3.1.4. Adaptive Tone Remapping

Assuming the image data to be normalized between 0 and 1, computations of the haze removal process usually produce results lying outside this range. The simple saturation arithmetic widely used in various algorithms reduces the dynamic range of the input image. Tarel et al. [12] first attempted to solve this problem by employing the tone remapping operation. However, their method operates solely on the luminance channel, which could induce color artifacts. In ICAP, we exploited a more sophisticated algorithm called adaptive tone remapping. It was first proposed by Cho et al. [39] to execute both luminance enhancement and color emphasis according to the following equations.

(8) $$\begin{array}{cc} & EL\left(x\right)=L\left(x\right)+G_{L}\left(x\right)W_{L}\left(x\right), \end{array}$$

(9) $$\begin{array}{cc} & EC\left(x\right)=C\left(x\right)+G_{C}\left(x\right)W_{C}\left(x\right)+0.5, \end{array}$$

where *L* denotes the input luminance, EL denotes the enhanced luminance, $G_{L}$ denotes the luminance gain, and $W_{L}$ denotes the adaptive luminance weight. The variables in Equation (), which gives the rule for color emphasis, can be interpreted similarly. The constant of 0.5 is an offset since the chrominance is zero-centered due to subtracting by 0.5 in advance. Interested readers are referred to Cho et al. [39] for a detailed description and computational formulas.

### 3.2. Atmospheric Light Estimation and Compensation Scheme for False Enlargement of White Objects

Existing algorithms that estimate atmospheric light usually suffer from two main problems: high computational costs and false localization of the light source. The method employed by He et al. [11] is a prime example. The top 0.1% brightest pixels in the dark channel, i.e., those corresponding to the most opaque region of an image, are first selected by the method. Among them, the pixel with the highest intensity in the input image is then singled out as a representative of the atmospheric light. This approach comprises expensive computations such as sorting the dark channel and searching over the selected pixels for the highest intensity. Plus, it undoubtedly fails in scenes containing white objects, because white pixels of normalized values (1,1,1) always stand out as the atmospheric light. The previously proposed ICAP [36] used the quad-decomposition method to avoid the high computational cost and false localization of the light source. In this method, the input image’s luminance is preprocessed by a minimum filter to reduce white objects’ influence. It is then divided into quarters, and the division is repeated in the quarter with the highest average intensity. The decomposition is continued until the quarter’s size is less than a predetermined value. In this final quarter, the pixel with the smallest Euclidean distance to the white point in the RGB space represents the atmospheric light.

**Figure 5:**
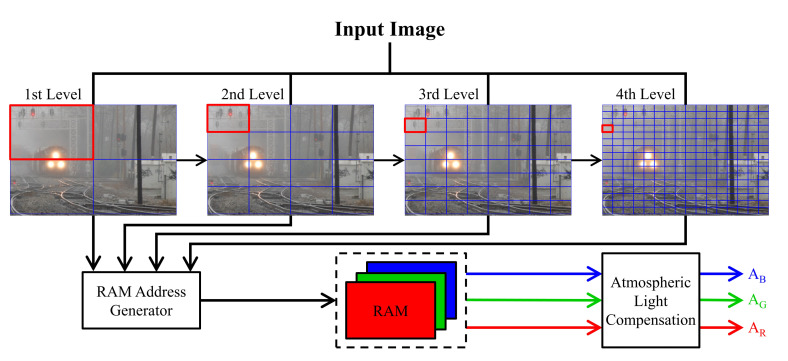
Parallel computing scheme for atmospheric light estimation and compensation.

**Figure 6:**
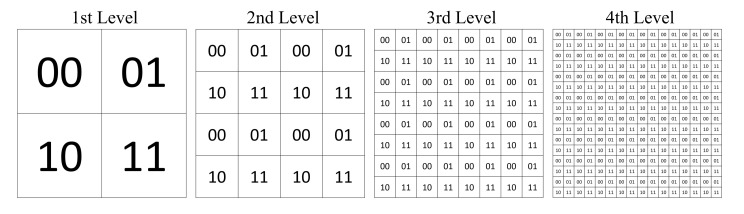
Example of labeling scheme for quarters at each level of decomposition.

However, from a hardware designer’s point of view, the quad-decomposition algorithm appears unattractive because of multiple image buffers’ requirement in its implementation. This paper aims to design a real-time hardware accelerator, and we accordingly propose an image buffer-free version of the quad-decomposition method. The preprocessing step with a minimum filter is retained without changed as it is computationally efficient and beneficial to reducing the influence of white objects. The decomposition step is modified following the procedure illustrated in Figure 5, where the number of decompositions is determined in advance, e.g., four in this case. At each level, the total number of decomposed image patches is an exponent of four, and each set of four individual local patches are labeled using ‘00’, ‘01’, ‘10’, and ‘11’. For example, at the second level, the number of patches is $4^{2}=16$, and there are four groups of ‘00’, ‘01’, ‘10’, and ‘11’ patches, as illustrated in Figure 6. The four levels are processed concurrently, and each of them outputs a label representing a selected patch. Meanwhile, 256 candidates for the atmospheric light corresponding to the 256 patches (=4${}^{4}$) comprising the fourth level are calculated and stored in three small RAMs. Then, by combining the four output labels into an 8-bit address, the atmospheric light can be easily read out from the memories.

**Figure 7:**
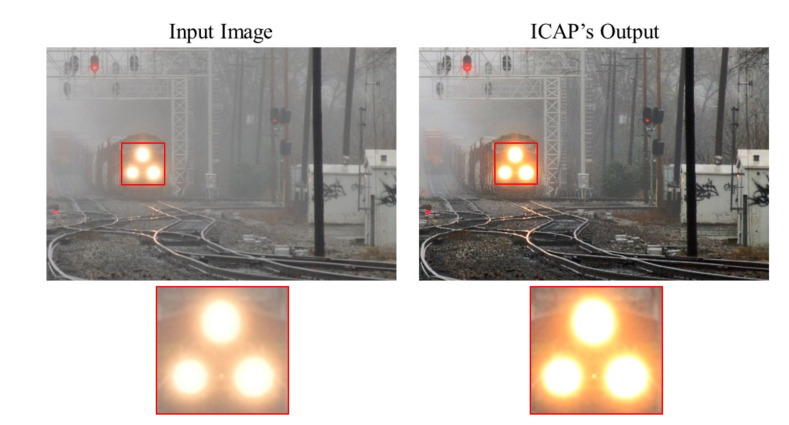
False enlargement problem shown by the train’s headlights.

**Figure 8:**
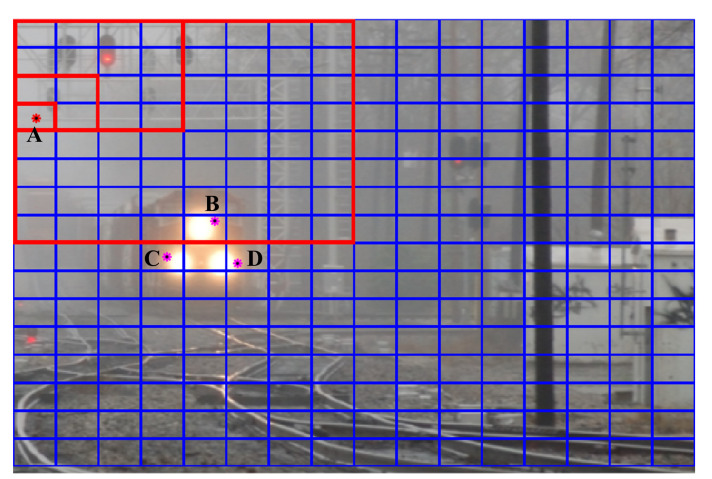
(A) Atmospheric light pixel and (B, C, and D) pixels surrounding the train’s headlight.

A post-dehazing false enlargement of white objects is a common problem affecting several haze removal algorithms, e.g., ICAP, as depicted in Figure 7. This paper represents the first attempt to address this problem. In the cropped region highlighted in red, the train’s headlight has mistakenly become larger after haze removal. The underlying cause of this is as follows. If the atmospheric light of lower intensity compared to specific image pixels, haze removal increases their intensity values instead of reducing them, which is evident from Equation (3). As a result, pixels surrounding the train’s headlight, which are of higher intensity than the atmospheric light, according to Figure 8 and Table 1, appear brighter after haze removal, causing the false enlargement.

**Figure 9:**
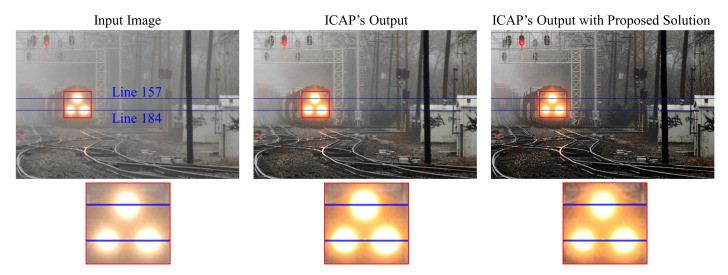
Result of applying the proposed atmospheric light compensation scheme.

**Figure 10:**
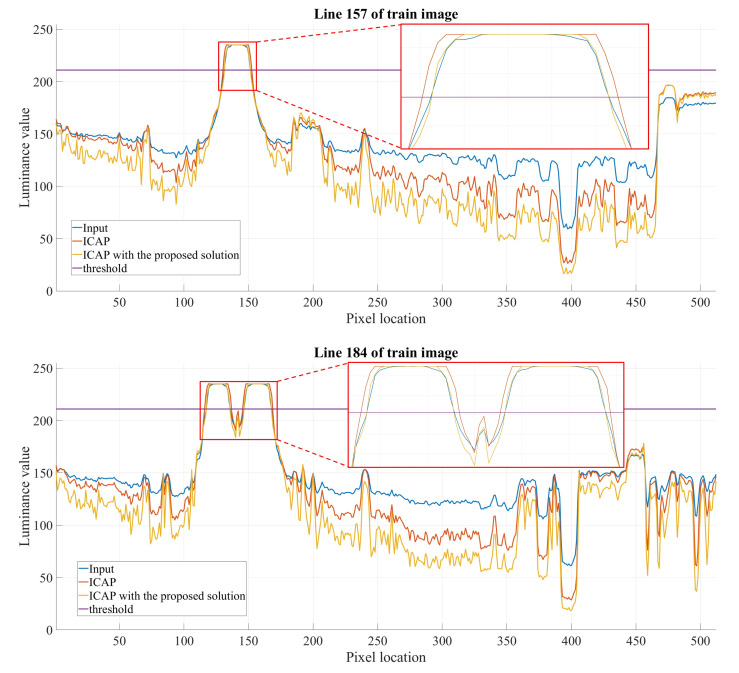
One-dimensional cross sections of the train’s headlights.

To overcome the false enlargement problem, we propose a compensation scheme that scales up the atmospheric light based on the difference between its channel-wise maximum and the maximum pixel intensity, using the following equation.

(10) $$\hat{A}=A+\omega_{A}\left\{max_{\Psi }\left[max_{c\in \{R,G,B\}}\left(I^{c}\right)\right]-max_{c\in \{R,G,B\}}\left(A^{c}\right)\right\},$$

where $\hat{A}$ denotes the compensated atmospheric light, Ψ denotes the entire image domain, and $\omega_{A}$ denotes the user-defined parameter controlling the compensation amount. When the input image contains a single light source, the compensation term is zero since the estimated atmospheric light is the brightest pixel. Conversely, when the input image contains multiple light sources, the estimated atmospheric light might not be as bright as other objects. Hence, the compensation term is necessary to avoid the false enlargement of white objects. The result presented in Figure 9 demonstrates that the false enlargement problem is successfully resolved by applying the proposed compensation scheme. Moreover, the one-dimensional cross-sections of the train’s headlights (i.e., lines 157 and 184 in Figure 9) depicted in Figure 10 and the measured diameters recorded in Table 2 quantitatively verify the effectiveness of the proposed solution in preventing the false enlargement problem. The straight purple line in Figure 10 denotes the reference luminance value of 211 during the measurement of the diameters of the train’s headlights.

### 3.3. Experimental Validation

#### 3.3.1. Quantitative Evaluation

This section evaluates the proposed algorithm against five benchmark approaches, including those proposed by He et al. [11], Tarel et al. [12], Zhu et al. [13], Kim et al. [27], and Ngo et al. [36] on both synthetic and real image datasets. FRIDA2 [40] is used as the synthetic image dataset, consisting of 66 clear images and 264 corresponding hazy images pertaining to four different haze types—homogeneous, heterogeneous, cloudy homogeneous, and cloudy heterogeneous. Computer graphics generate each of the images for advanced driver-assistance systems. The second synthetic dataset is D-HAZY [41], comprising more than 1400 indoor images and their corresponding scene depths captured via Microsoft’s Kinect sensor. Koschmieder’s law is then in order for synthesizing the corresponding hazy images. IVC [42], O-HAZE [43], and I-HAZE [44] are real image datasets considered. IVC consists of 25 real hazy images of various subjects, including landscapes, animals, humans, and plants. O-HAZE contains 45 pairs of outdoor hazy and haze-free images, while I-HAZE is composed of 30 pairs of indoor hazy and haze-free images. Haze was added to the images in the O-HAZE and I-HAZE datasets by using a specialized vapor generator.

For image datasets with available ground-truths, structural similarity (SSIM) [45], tone-mapped image quality index (TMQI) [46], feature similarity extended to color images (FSIMc) [47], and FADE [30] are the assessment metrics. In contrast, the rate of new visible edges (e) and the quality of contrast restoration (r) proposed by Hautiere et al. [48] are used alongside FADE for image datasets that do not contain ground-truth references. Assuming that X and Y denote two image luminance signals, the SSIM measure between them is calculated as follows.

(11) $$SSIM\left(X,Y\right)=\frac{\left(2\mu_{x}\mu_{y}+C_{1}\right)\left(2\sigma_{xy}+C_{2}\right)}{\left(\mu_{x}^{2}+\mu_{y}^{2}+C_{1}\right)\left(\sigma_{x}^{2}+\sigma_{y}^{2}+C_{2}\right)},$$

where $\left(\mu_{x},\mu_{y}\right)$ and $\left(\sigma_{x},\sigma_{y}\right)$ denote the local mean and local standard deviation of (X,Y), respectively; $\sigma_{xy}$ denotes the correlation coefficient between $\left(X-\mu_{x}\right)$ and $\left(Y-\mu_{y}\right)$, and $\left(C_{1},C_{2}\right)$ denote positive constants that prevent the values of $\left(\mu_{x}^{2}+\mu_{y}^{2}\right)$ and $\left(\sigma_{x}^{2}+\sigma_{y}^{2}\right)$ from approaching too close to zero. SSIM varies between 0 and 1, and a higher value indicates that the compared image structurally resembles the ground-truth reference to a greater extent.

TMQI is a measure that evaluates the multi-scale structural similarity in combination with the naturalness of images. It is given by Equation (12), where S(X,Y) denotes the multi-scale structural fidelity, N(X,Y) denotes the statistical naturalness measure, the parameter 0≤a≤1 controls the relative importance of S(X,Y) and N(X,Y), and α and γ are used to adjust their respective sensitivities. The value of TMQI ranges between 0 and 1, and a higher score is more favorable to visibility restoration tasks.

(12) $$TMQI\left(X,Y\right)=a\cdot S{\left(X,Y\right)}^{\alpha }+\left(1-a\right)\cdot N{\left(X,Y\right)}^{\gamma }.$$

As both SSIM and TMQI operate solely on the image luminance channel, FSIMc is additionally adopted to conduct a more thorough evaluation. Zhang et al. [47] developed FSIMc based on the observation that low-level features, including phase congruency, image gradient magnitude, and chrominance similarity, exert a significant influence on the human perception of images. FSIMc is computed using the following equation.

(13) $$FSIMc\left(X,Y\right)=\frac{\sum_{i\in \Psi }S_{L}\left(i\right)\cdot S_{C}{\left(i\right)}^{\nu }\cdot PC_{m}\left(i\right)}{\sum_{i\in \Psi }PC_{m}\left(i\right)},$$

where X and Y henceforth denote color images, Ψ denotes the entire image domain, $S_{L}$ denotes the combined similarity, $S_{C}$ denotes the chrominance similarity, $PC_{m}$ denotes the weighting coefficient, and ν denotes a positive constant that controls the importance of the chrominance. FSIMc also varies between 0 and 1, where higher values induce a better performance.

Concerning the evaluation metrics for datasets without reference ground truths, Hautiere et al. [48] proposed two indicators based on restored edges visible in the output but not in the input. They are given by the following equations.

(14) $$\begin{array}{cc} & e=\frac{n_{r}-n_{o}}{n_{o}}, \end{array}$$

(15) $$\begin{array}{cc} & r=exp\left[\frac{\sum_{i\in \Phi }log\left(r_{i}\right)}{n_{r}}\right], \end{array}$$

where $n_{r}$ and $n_{o}$ denote the numbers of sets of visible edges in the restored image and the original image, respectively, and $r_{i}$ denotes the ratio indicating the improvement in visibility with respect to the set of visible edges, Φ. Both e and r are directly proportional to the quality of image enhancement. However, it is worth noticing that these indicators are susceptible to noise. Therefore, it is advisable to employ them together with a qualitative assessment for accurate judgment. FADE is another evaluation measure for images without ground truths, and it has been discussed previously in Section 2.2. As FADE proportionally represents the image’s haze density, smaller FADE values correspond to better haze removal algorithms. However, FADE suffers from the same problem as e and r because it does not take the structural information into account, leading to a phenomenon that overly dehazed images with noticeable loss of details surprisingly result in smaller FADE scores. Thus, it is also advisable to employ FADE in conjunction with other metrics or a qualitative evaluation to guarantee dehazing assessment reliability.

Table 3 and Table 4 present the average SSIM, TMQI, FSIMc, and FADE scores on the FRIDA2 and D-HAZY datasets, respectively. The boldface numbers represent the best results. On the FRIDA2 dataset, the proposed method exhibits the best dehazing performance in terms of TMQI and FSIMc and the second-best under SSIM and FADE. Since FRIDA2 comprises images of road scenes solely, the atmospheric light compensation scheme for preventing white objects’ false enlargement has little effect in this case. On the D-HAZY dataset, the proposed algorithm is observed to perform the best in terms of FADE and the third-best in terms of SSIM, TMQI, and FSIMc. This observation can be attributed to the fact that D-HAZY consists of daylight indoor images of similar scenes, while the proposed method is tuned to achieve acceptable performance in most circumstances. Overall, the experimental results are consistent with those reported by Ancuti et al. [41]. Their results also demonstrated that the algorithm proposed by He et al. [11] exhibited the best dehazing performance on the D-HAZY dataset.

Table 5, Table 6 and Table 7 display the quantitative evaluation results on the IVC, O-HAZE, and I-HAZE datasets, respectively. On IVC, the algorithm proposed by Tarel et al. [12] performs the best in terms of e and r because of the metrics’ shortcoming of misinterpreting noise as visible edges. Hence, the primary contributors to its high e and r scores are halo artifacts and background noise. The method proposed by Kim et al. [27] is developed based on the one by Tarel et al. [12] to suppress halo artifacts but not background noise, therein lies the cause of smaller e and r scores. Our previous work, which is the method developed by Ngo et al. [36], eliminated background noise, achieving smaller e and r scores than the two methods mentioned earlier. The algorithm proposed in this paper, equipped with the atmospheric light compensation scheme, furthers the improvement to achieve better results in terms of e and FADE. It is observed to be the best performing method on the IVC dataset. On the O-HAZE dataset, the proposed method shares the best performance with that proposed by He et al. [11]—whereas their algorithm exhibits the best scores in terms of SSIM and FSIMc, ours exhibits the best dehazing performance with respect to TMQI and FADE. The proposed approach achieves even more impressive results on I-HAZE dataset, as illustrated by the highest SSIM, TMQI, and FSIMc scores.

#### 3.3.2. Qualitative Evaluation

**Figure 11:**
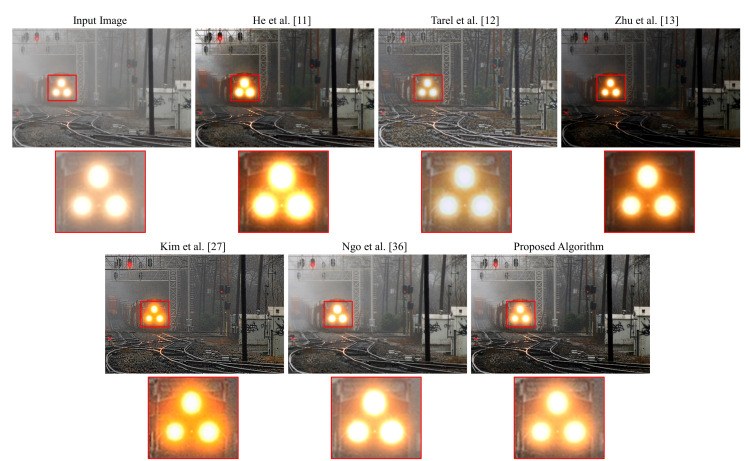
Qualitative assessment of the outputs produced by different algorithms on an image of a train.

Figure 11 depicts a real hazy photograph of a train. We use this image to qualitatively assess the dehazing performance of both the proposed algorithm and the five benchmark methods. It is evident that the methods proposed by He et al. [11] and Ngo et al. [36] suffer from the false enlargement problem. The cause underlying this visual artifact was discussed in Section 3.2. In contrast, this issue is not apparent in the output image produced by the methods proposed by Tarel et al. [12] and Kim et al. [27] since the atmospheric light is always the maximum value of (1,1,1) in these methods. However, halo artifacts and background noise are noticeable. The algorithm proposed by Zhu et al. [13] produces an over-dehazed image due to the use of a fixed lower bound for the transmission map. The algorithm proposed in this paper generates the most satisfactory result without halo artifacts, background noise, false enlargement, and over-removal of haze.

**Figure 12:**
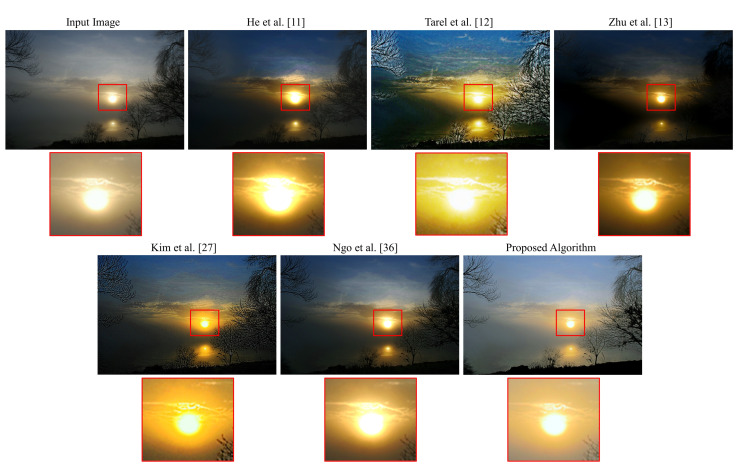
Qualitative assessment of the outputs produced by different algorithms on an image of a sunset.

**Figure 13:**
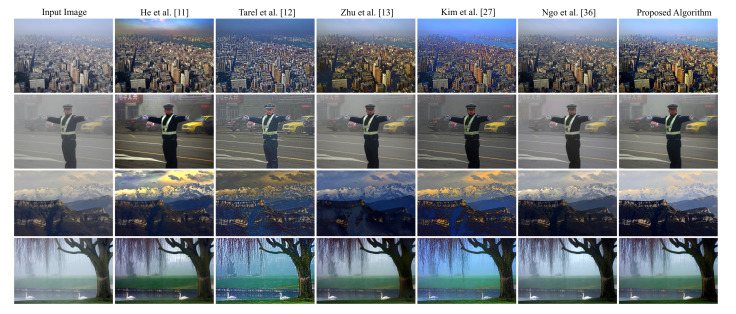
Qualitative assessment of the outputs produced by different algorithms on other real-world images.

Figure 12 illustrates a real hazy nocturnal scene of a sunset. It is apparent that the proposed algorithm produces a result that favors the human perception of image quality, as it removes haze without introducing any unpleasant side-effects. As in the previous case, the false enlargement problem is noticeable in the outputs obtained via the methods proposed by He et al. [11] and Ngo et al. [36]. The method proposed by Tarel et al. [12] suffers from severe halo artifacts near the fine details of the tree’s twigs. The method proposed by Zhu et al. [13] overly dehazes the image, producing a result that is too dark, completely obscuring the tree’s twigs. Other examples, supporting the conclusion that the proposed algorithm outperforms the five benchmark methods, can be found in Figure 13.

## 4. A 4K-Capable Hardware Accelerator

In general, algorithms are useful if they can find their applications in popular real-world systems. Specifically, for an image processing algorithm to be a part of real-time systems, such as surveillance cameras, it is required to satisfy a minimum processing rate of 25 or 30 frames per second (fps). This requirement depends on whether the employed color encoding system is Phase Alternation by Line (PAL) or National Television System Committee (NTSC) [49], respectively. Table 8 presents the processing times achieved by the proposed method and the five benchmark methods. The data demonstrate that none of the methods deliver the required processing speed. All six algorithms were programmed in MATLAB R2019a and tested on a computer with Intel Core i9-9900K (3.6 GHz) CPU, 64 GB RAM, and NVIDIA TITAN RTX GPU. For the smallest image resolution of VGA (Video Graphics Array, 640×480), the fastest method was the one proposed by Kim et al. [27], which was only able to process 6.25 fps (=1/0.16). As the image size increases, the processing rates decrease dramatically, and the maximum attainable speed for 4K resolution (4096×2160) is merely 0.21 fps (≈1/4.81). These observations suggest that the software implementation is unable to put visibility restoration algorithms into practical use. We present a hardware accelerator capable of processing images in 4K resolution at 30.7 fps to address this issue.

### 4.1. Overall Architecture

**Figure 14:**
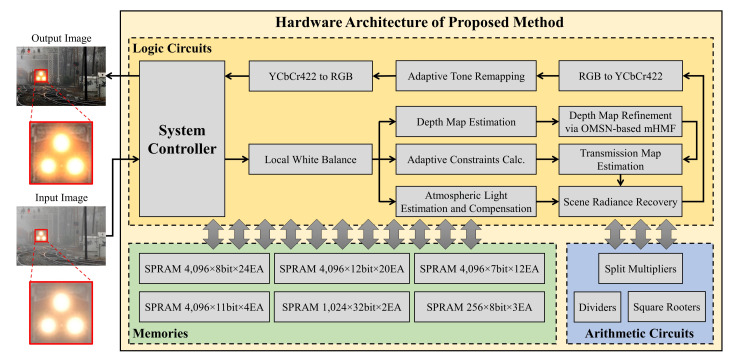
Overall architecture of the hardware accelerator for the proposed algorithm.

Figure 14 depicts the overall hardware architecture of the proposed method. The system controller is responsible for input-output operations of the image data, and it employs a double-buffering scheme with separate read/write buffers to avoid data bottleneck. The local white balance module processes the input RGB image to remove any unrealistic color casts and transmits the data to three modules—depth map estimation, adaptive constraints calculation, and atmospheric light estimation and compensation—in parallel. The depth map estimation module performs the following series of operations:

- RGB-to-HSV conversion,
- low-pass filtering on the saturation channel to suppress background noise,
- and depth map estimation using Equation (4) with predetermined parameters via MLE.

The modified hybrid median filter, which is realized by a novel hardware architecture named optimized merging sorting network, then refines the estimated depth map. The following subsection will delve deeply into this novel hardware architecture. The adaptive constraints calculation module computes the two adaptive constraints presented in Equation (5). Simultaneously, the atmospheric light estimation and compensation module identifies the compensated lightness pixel via the parallel scheme discussed in Section 3.2. Section 4.3 will set this module out in greater detail. The transmission map is easily calculated based on the refined scene depth and the two constraints since the exponential function $t\left(x\right)=e^{-\beta d(x)}$ can be efficiently realized using a look-up table (LUT). Subsequently, the scene radiance recovery module calculates the adaptive weight $\omega_{t}\left(x\right)$ defined by Equation (6) and recovers the scene following Equation (7). Finally, the adaptive tone remapping module performs dynamic range expansion to enhance the recovered image, in which RGB-to-YCbCr422 and YCbCr422-to-RGB modules are deployed as color format converters to facilitate its computations. Arithmetic circuits, including split multipliers, dividers, and square rooters, are separated from the main computations to favor the automated place-and-route procedure. Likewise, the employed block memories are also segregated from the logic circuits. The proposed hardware accelerator uses three 256×8-bit memories to store the atmospheric light pixel candidates, as mentioned in Section 3.2. Two 1024×32-bit memories are used to calculate the requisite histogram for the adaptive tone remapping post-processing, while other memories are used as line memories to filter operations. It is worth noticing that the proposed design does not use any of the memories as image buffers.

### 4.2. Optimized Merging Sorting Network-Based Architecture for the Modified Hybrid Median Filter

**Figure 15:**
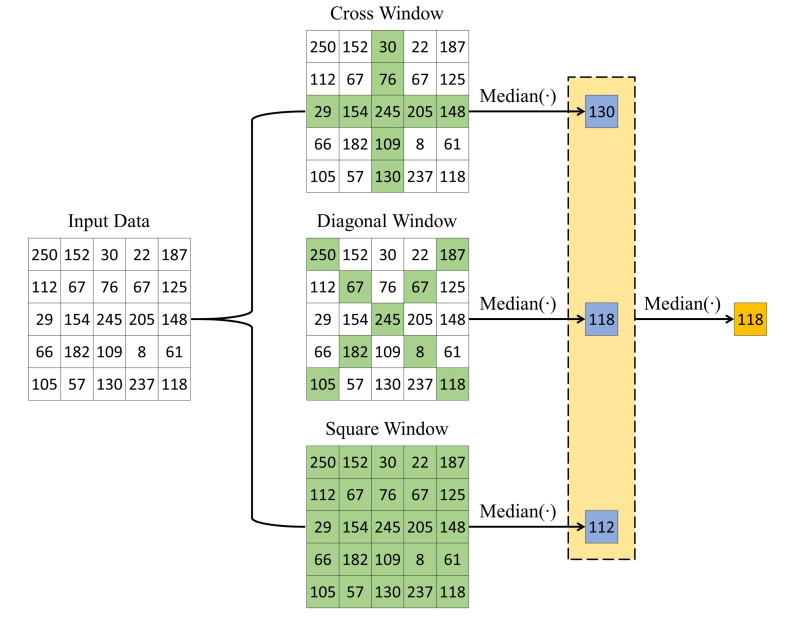
Example of the operation of the modified hybrid median filter (mHMF).

The modified hybrid median filter (mHMF) is employed in the proposed algorithm to refine the estimated depth map. Based on the observation that the scene depth is predominantly smooth except for discontinuities such as objects’ contours, the application of the standard median filter (SMF), as in the method proposed by Tarel et al. [12], leads to the problem of smoothing image edges, subsequently inducing the halo artifacts discussed in Section 3.3.2. mHMF overcomes this problem by using the cross and diagonal windows in combination with the traditional square window. It identifies three median values corresponding to three windows and then calculates their median as the final result. Figure 15 demonstrates the process of mHMF on specific input data.

The mHMF exhibits better edge-preserving characteristics at the cost of more expensive computations, giving rise to its burdensome hardware implementation. Our previous work in [18] presented the first attempt to develop a fast and compact architecture based on Batcher’s parallel sorting network (BSN). mHMF with a N×N window is decomposed into four SMFs, including an $N^{2}$-input filter for the square window, two (2N−1)-input filters for the cross and diagonal windows, and a 3-input filter to select the final result. Since the median identification procedure essentially comprises sorting input data and separating the median value, the median filter design can be further simplified to the design of a sorting network, which comprises a set of compare-and-swap (CS) operations connected with a fixed configuration of interconnections. Therefore, the use of BSNs to construct the mHMF results in a fast and compact architecture. However, it suffers from the significant problem of repeated use of specific pixels within the filtering window. The reason is the use of three separate SMFs for three different types of windows. For example, BSN-based mHMF uses the central pixel thrice and other pixels that lie on the cross and diagonal lines twice. This issue increases the fan-out of related logic gates, resulting in the elongation of the signal propagation delay. The following equation, presented in [50], is employed to quantify the influence of fan-out on propagation delay.

(16) $$t_{D}=m+n\cdot SL,$$

where $t_{D}$ denotes the propagation delay of a logic gate, (m,n) denotes a pair of constants characterizing its timing behavior, and SL denotes the standard load connected to its output. When an output signal from a logic gate is wired to another digital circuit, it is convenient to model the target circuit as a capacitive load. Thus, if a signal is wired to several different circuits, the output capacitive load of a logic gate producing that signal is increased in an additive manner, causing the elongation of the signal propagation delay according to Equation (16).

Based on the aforementioned investigation, it is evident that addressing the problem of high fan-out guarantees shortened propagation delay, i.e., an improvement in the throughput. Therefore, we propose a new architecture called an optimized merging sorting network (OMSN), which uses pixels within the filtering window exactly once. OMSN is based on the observation that it is unnecessary to sort the pixels within the three windows separately to identify the final median. Instead, it suffices to follow the following procedure.

1. Only sorting pixels within one of the two small windows, e.g., the cross window, to identify the corresponding median.
2. For the diagonal window, sorting corresponding pixels except for the central one and then merging them with the delayed central pixel to identify the median.
3. For the square window, only sorting those pixels that have not been sorted during the previous two steps and merging them with the two sorted sequences to identify the corresponding median.
4. Lastly, selecting the final median from the medians corresponding to the three windows.

**Figure 16:**
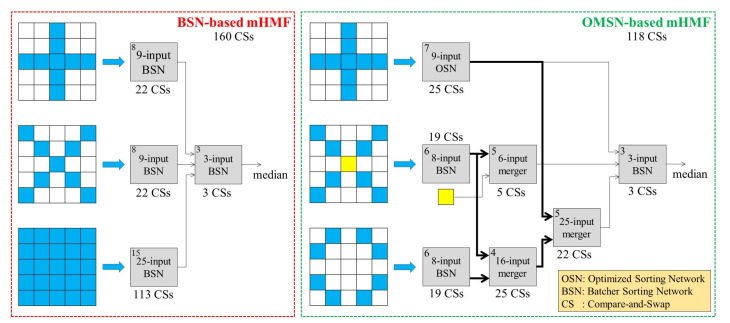
Previously developed and the newly proposed architectures for a 5×5 mHMF.

Figure 16 depicts the BSN-based and the OMSN-based architectures for a 5×5 mHMF. The yellow cell represents the central pixel’s delayed value, and the abbreviation OSN denotes the optimized sorting network presented in [51]. For each module, the top-left number denotes the corresponding latency in terms of clock cycles, and the under or above number denotes the constituent CS operations. The 9-input BSN and the 9-input OSN are fundamentally different. The former comprises 22 CSs because it eliminates the CSs that are not pertinent to the median identification procedure. In contrast, the latter (i.e., 9-input OSN) needs to retain all of its inputs to identify the median corresponding to the square window, giving rise to the difference in the number of CSs between the two approaches. However, the difference between their latencies demonstrates that the 9-input OSN is superior to the 9-input BSN. The BSN-based mHMF architecture comprises 160 CSs and exhibits a latency of 18 clock cycles. In contrast, the proposed OMSN-based mHMF architecture consists of merely 118 CSs. Notwithstanding the same latency of 18 clock cycles, the proposed design is faster since it reduces the clock period by precluding the high fan-out problem.

In order to validate the aforementioned claims, mHMFs with 5×5 and 7×7 windows were implemented on a system-on-a-chip (SoC) evaluation board [52] using the Verilog hardware description language (IEEE Standard 1364-2005) [53]. The corresponding hardware synthesis results were summarized in Table 9, in which slice registers and slice LUTs represent logical gate regions, and RAM36E1/FIFO36E1s represent memory regions. The ‘Used as Memory’ category denotes the number of LUTs that can be synthesized as distributed memories, while the block memories are mapped to RAM36E1/FIFO36E1s. The synthesis data demonstrate that the number of used registers and LUTs is reduced significantly using the proposed OMSN-based architecture. More specifically, the reduction rates are 17.5% and 18.0% for a 5×5 mHMF, and 16.1% and 13.5% for a 7×7 mHMF. The number of requisite RAM36E1/FIFO36E1s is equal for both models as they are used to realize line memories, which are 4 and 6 for the window sizes of 5×5 and 7×7, respectively. Finally, by resolving the high fan-out problem, the proposed OMSN-based design improves the throughput by approximately 10% for both the 5×5 and 7×7 mHMF.

### 4.3. Atmospheric Light Estimation and Compensation

**Figure 17:**
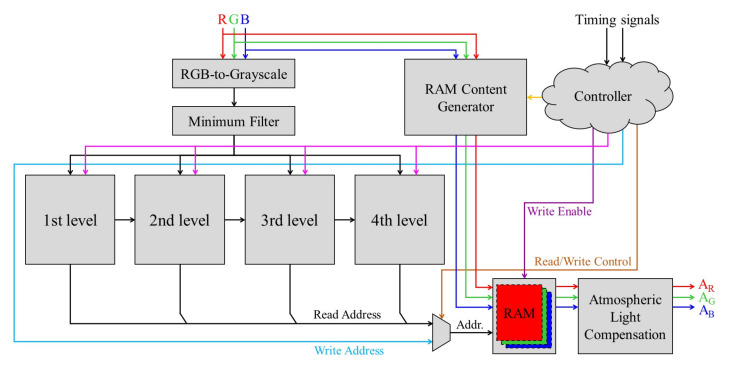
The proposed hardware architecture for atmospheric light estimation and compensation.

Figure 17 depicts the hardware architecture used for atmospheric light estimation and compensation. The input RGB image data first undergo a conversion to extract the grayscale channel, which in turn undergoes the minimum filter to reduce white objects’ influence on the estimation process’s accuracy. Four decomposition levels accept the filtered grayscale channel in parallel, and each module computes the corresponding 2-bit index ‘00’, ‘01’, ‘10’, or ‘11’ corresponding to the image patch with the highest average intensity. The index information is passed down across all levels between the second and the fourth because the chosen image patch at each level must necessarily lie within the image patch selected at the preceding level. Additionally, the four indices are combined to form an 8-bit read address to identify the lightness pixel from the memories. Simultaneously, RGB data also undergo the RAM content generator module, which calculates all 256 candidates for the atmospheric light. These candidates, coupled with timely signals including write address, read/write control, and write enable, are stored in memories. The controller module receives timing signals of input RGB data, i.e., horizontal and vertical active video signals, and is responsible for the proper functioning of all other modules. Then, the estimated lightness pixel is read out from the memories and is made to undergo the compensation procedure described by Equation (10). While the channel-wise maximum operations, $max_{c\in \{R,G,B\}}\left(I^{c}\right)$ and $max_{c\in \{R,G,B\}}\left(A^{c}\right)$, are easily implemented, the maximum operation across the entire image domain $max_{\Psi }\left(\cdot \right)$ generally requires an image buffer. However, by exploiting the high similarity between successive video frames, a viable alternative is to identify the maximum corresponding to each current frame and apply it to the next frame. As a result, the necessity of an image buffer is completely eliminated from the proposed architecture depicted in Figure 17.

### 4.4. Hardware Verification

**Figure 18:**
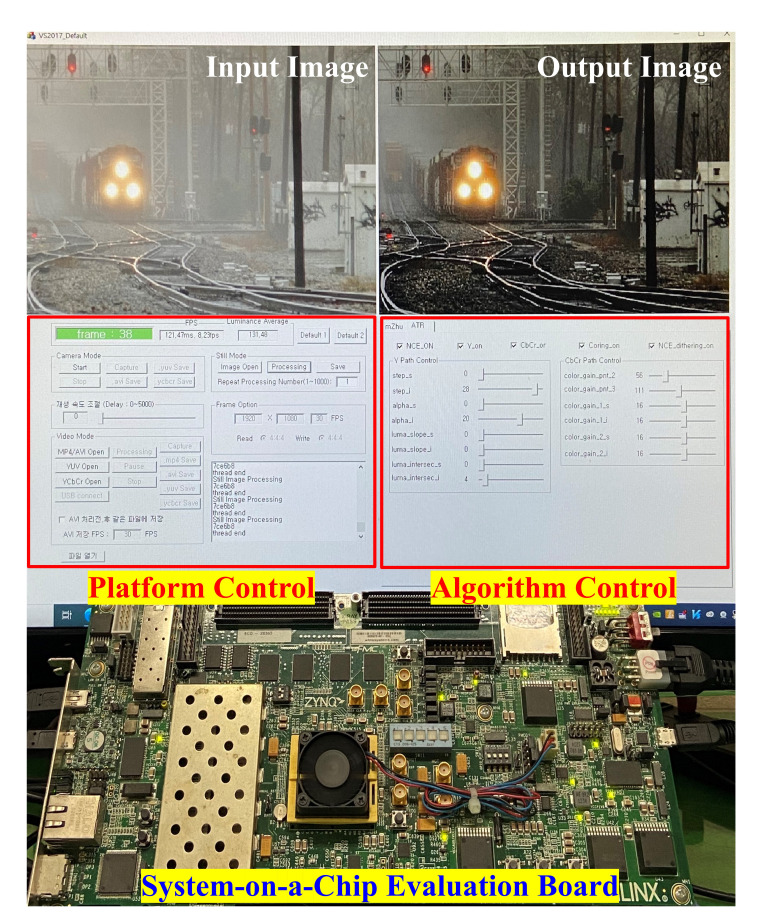
Hardware verification using a system-on-a-chip evaluation board.

The SoC evaluation board mentioned in Section 4.2 is used for hardware verification at this stage. It includes a Field Programmable Gate Array (FPGA) chip, dual ARM Cortex-A9 core processors, 1 GB DDR3 memory, and 1 GB DDR3 SODIMM. A C/C++ platform is developed on a host computer to provide input data to and read processed data from the SoC board. The top and middle thirds of Figure 18 depict the platform, while the bottom third depicts the board. Users can select the input data from various sources, such as still images, real-time videos from a camera, or videos stored on the host computer, via the platform control. On the other hand, the algorithm control includes several slide bars and check-boxes, which can tune input parameters before sending them to the implemented design on the evaluation board. Input and output data are displayed side-by-side, as depicted in the top third of Figure 18. The output image on the right depicts the result obtained from the SoC evaluation board. Users can also select one of two video saving modes—output only and input-output (side-by-side)—using the platform’s buttons. This C/C++ platform is used to verify the performance of the proposed hardware accelerator. Based on the top third of Figure 18, it is evident that the false enlargement in the image of the train was effectively surmounted, which is consistent with the result illustrated previously in Figure 11. Moreover, the overall visibility was significantly improved, which is apparent based on the observation of the train’s cars. This visibility improvement is primarily attributed to the post-processing application of adaptive tone remapping, while the success in dealing with the false enlargement problem is attributed to the proposed compensation scheme presented in Section 3.2.

Table 10 summarized the detailed hardware synthesis result corresponding to the proposed visibility restoration algorithm. Our design used 57,848 registers, 53,569 LUTs, 58 RAMB36E1s, and 25 RAMB18E1s, which occupied 13.23%, 24.51%, 10.64%, and 2.29% of available resources on the FPGA chip, respectively. The fastest attainable processing rate was 271.67 MHz, or equivalently, 271.67 Mpixel/s. Based on this information, the maximum processing speed (MPS) in terms of fps can be calculated as follows.

(17) $$MPS=\frac{f_{max}}{\left(W+HB)(H+VB\right)},$$

where $f_{max}$ denotes the maximum operating frequency; *W* and *H* denote the width and the height of the input frame, respectively; and HB and VB denote the horizontal and vertical blank periods. In this study, the hardware accelerator was designed to function properly while minimizing the number of blank periods corresponding to one pixel and one image line. The MPSs achieved for different video resolutions, as recorded in Table 11, demonstrated that the proposed design is capable of processing the maximum video resolution of DCI 4K at 30.7 fps. In particular, the number of clock cycles required by the proposed algorithm to process one frame is 8,853,617 (=4097 × 2161). Substituting this value into Equation (17) yields an MPS of 30.7 (≈271.67 × 10${}^{6}$/8,853,617). Therefore, the proposed hardware accelerator is verified to be highly appropriate for real-time applications requiring fast processing of high-quality images.

Table 12 summarized the results of a comparative evaluation of the proposed implementation in the context of those of other hardware designs. Park et al. [54] developed a fast implementation of DCP by reducing the complexity of the atmospheric light estimation procedure. Although their design exhibited a maximum operating frequency of 88.70 MHz, it could only handle the fixed frame sizes of 320×240, 640×480, and 800×600. Thus, it is solely capable of processing images of the Super VGA (SVGA) resolution. Moreover, except for the number of used registers, it requires more of every other resource than the algorithm proposed in this paper—in terms of LUTs, digital signal processing slices (i.e., DSPs), and memories. Ngo et al. [18] presented a direct implementation of the algorithm developed by Kim et al. [27] using fewer memories than the proposed accelerator. However, this difference is because the proposed algorithm employs more filtering operations than the one developed by Kim et al. [27]. As discussed in Section 3.3, this enables better dehazing performance. Furthermore, the accelerator proposed in this study is more efficient in terms of the used registers and LUTs. Additionally, although both designs can handle DCI 4K resolution, our algorithm delivers faster speed and is preferable. Furthermore, the design implemented by Ngo et al. [18] is only compatible with the PAL color encoding system, while ours is compatible with both PAL and NTSC standards.

## 5. Conclusions

In this paper, we presented a machine learning-based visibility restoration algorithm and its corresponding 4K-capable hardware accelerator. The proposed algorithm is an improvement on our previous work based on the color attenuation prior. We devised effective solutions to solve the common problems observed in existing algorithms, such as background noise, color distortion, reduced dynamic range, and false enlargement of white objects. We also exploited the enhanced equidistribution to prepare a more reliable training dataset, used to estimate parameters of the depth estimating model via supervised learning using the maximum likelihood estimates technique. Notably, the proposed approach represents the first attempt to address the false enlargement of white objects. By identifying the cause of this problem to be the difference between the atmospheric light and bright pixels surrounding white objects, we proposed a compensation scheme to deal with the unpleasant visual effects effectively. Experimental results proved the superiority of the proposed algorithm over the five benchmark methods, both quantitatively and qualitatively. The source code and datasets are publicly available for facilitating future research: https://datngo.webstarts.com/blog/.

It was discovered during the hardware implementation phase that the previously developed hardware architecture for the modified hybrid median filter suffers from a high fan-out problem. To rectify this, we proposed an optimized merging sorting network-based architecture as an efficient alternative and achieved a reduction in hardware use and an increase in throughput. Moreover, to eliminate the necessity for image buffers during the implementation of the quad-decomposition algorithm, we adopted a parallel computing scheme, which is highly beneficial for real-time processing. The hardware synthesis result demonstrated that the proposed design could handle a maximum DCI 4K resolution at 30.7 fps. Additionally, a comparative evaluation against two other designs further proved that our hardware accelerator is relatively efficient in terms of resource use and throughput, making it highly appropriate for a wide variety of real-time applications.

## Figures and Tables

**Table 1:** Red-Green-Blue (RGB) values of atmospheric light pixel and pixels surrounding the train’s headlight.

Pixel	RGB Values	
	Before Dehazing	After Dehazing
A	[0.7529, 0.7529, 0.7529]	[0.8000, 0.8000, 0.8000]
B	[0.9804, 0.7961, 0.6235]	[1.0000, 0.8549, 0.5020]
C	[0.9373, 0.7569, 0.5961]	[1.0000, 0.7804, 0.4627]
D	[0.9922, 0.8078, 0.6314]	[1.0000, 0.8784, 0.5176]

**Table 2:** Diameter of one-dimensional cross sections of the train’s headlight.

Line	Diameters (pixels)		
	Input Image	ICAP’s Output	ICAP’s Output with
			the Proposed Solution
157	20	22	20
184	Left = 18, Right = 21	Left = 21, Right = 23	Left = 18, Right = 21

**Table 3:** Average structural similarity (SSIM), tone-mapped image quality index (TMQI), feature similarity extended to color images (FSIMc), and fog aware density evaluator (FADE) scores on FRIDA2 dataset.

Method	Haze Type	SSIM	TMQI	FSIMc	FADE
He et al. [11]	Homogeneous	0.6653	0.7639	0.8168	1.0177
	Heterogeneous	0.5374	0.6894	0.7251	1.2793
	Cloudy Homogeneous	0.5349	0.6849	0.7222	1.2587
	Cloudy Heterogeneous	0.6500	0.7781	0.8343	1.0792
	Overall Average	0.5969	0.7291	0.7746	1.1587
Tarel et al. [12]	Homogeneous	0.7096	0.7259	0.7833	0.9307
	Heterogeneous	0.6970	0.7310	0.7725	1.4961
	Cloudy Homogeneous	0.6719	0.7312	0.7567	1.3583
	Cloudy Heterogeneous	0.7431	0.7373	0.8104	1.1021
	Overall Average	0.7054	0.7314	0.7807	1.2218
Zhu et al. [13]	Homogeneous	0.5651	0.7533	0.7947	0.5527
	Heterogeneous	0.5519	0.7254	0.7845	0.9599
	Cloudy Homogeneous	0.5310	0.7080	0.7764	0.8267
	Cloudy Heterogeneous	0.5412	0.7674	0.8117	0.6752
	Overall Average	0.5473	0.7385	0.7918	**0.7536**
Kim et al. [27]	Homogeneous	0.5949	0.7320	0.8048	0.9675
	Heterogeneous	0.6245	0.7037	0.7805	1.6836
	Cloudy Homogeneous	0.6124	0.7015	0.7751	1.5741
	Cloudy Heterogeneous	0.6078	0.7343	0.8135	1.0774
	Overall Average	0.6099	0.7179	0.7935	1.3256
Ngo et al. [36]	Homogeneous	0.7022	0.7475	0.8013	0.7825
	Heterogeneous	0.7089	0.7318	0.7919	1.1610
	Cloudy Homogeneous	0.6918	0.7268	0.7854	1.0711
	Cloudy Heterogeneous	0.7253	0.7539	0.8152	0.8895
	Overall Average	**0.7070**	0.7400	0.7984	0.9761
Proposed Algorithm	Homogeneous	0.7039	0.7491	0.8020	0.7856
	Heterogeneous	0.7046	0.7339	0.7918	1.1485
	Cloudy Homogeneous	0.6864	0.7288	0.7860	1.0522
	Cloudy Heterogeneous	0.7282	0.7538	0.8153	0.8834
	Overall Average	0.7058	**0.7414**	**0.7988**	0.9674

**Table 4:** Average SSIM, TMQI, FSIMc, and FADE scores on D-HAZY dataset.

Method	SSIM	TMQI	FSIMc	FADE
He et al. [11]	**0.8348**	0.8631	**0.9002**	0.7422
Tarel et al. [12]	0.7475	0.8000	0.8703	0.9504
Zhu et al. [13]	0.7984	0.8206	0.8880	0.9745
Kim et al. [27]	0.7520	**0.8702**	0.8590	0.8556
Ngo et al. [36]	0.7691	0.8165	0.8787	0.7420
Proposed Algorithm	0.7766	0.8373	0.8788	**0.7325**

**Table 5:** Average e, r, and FADE scores on IVC dataset.

Method	e	r	FADE
He et al. [11]	0.39	1.57	0.56
Tarel et al. [12]	**1.30**	**2.15**	0.53
Zhu et al. [13]	0.78	1.17	0.83
Kim et al. [27]	1.27	2.07	0.73
Ngo et al. [36]	1.11	2.03	0.50
Proposed Algorithm	1.16	2.03	**0.46**

**Table 6:** Average SSIM, TMQI, FSIMc, and FADE scores on O-HAZE dataset.

Method	SSIM	TMQI	FSIMc	FADE
He et al. [11]	**0.7709**	0.8403	**0.8423**	0.3719
Tarel et al. [12]	0.7263	0.8416	0.7733	0.4013
Zhu et al. [13]	0.6647	0.8118	0.7738	0.6531
Kim et al. [27]	0.4702	0.6509	0.6869	1.1445
Ngo et al. [36]	0.7322	0.8935	0.8219	0.3647
Proposed Algorithm	0.7520	**0.9017**	0.8212	**0.3612**

**Table 7:** Average SSIM, TMQI, FSIMc, and FADE scores on I-HAZE dataset.

Method	SSIM	TMQI	FSIMc	FADE
He et al. [11]	0.6580	0.7319	0.8208	0.8328
Tarel et al. [12]	0.7200	0.7740	0.8055	**0.8053**
Zhu et al. [13]	0.6864	0.7512	0.8252	1.0532
Kim et al. [27]	0.6424	0.7026	0.7879	1.7480
Ngo et al. [36]	0.7600	0.7892	0.8482	1.1277
Proposed Algorithm	**0.7781**	**0.8122**	**0.8655**	0.8556

**Table 8:** Processing time in seconds of haze removal algorithms for different image resolutions.

Method∖Image Size	640 × 480	800 × 600	1024 × 768	1920 × 1080	4096 × 2160
**He et al. [11]**	12.64	19.94	32.37	94.25	470.21
**Tarel et al. [12]**	0.28	0.59	0.76	1.51	9.02
**Zhu et al. [13]**	0.22	0.34	0.55	1.51	6.39
**Kim et al. [27]**	0.16	0.29	0.43	1.01	4.81
**Ngo et al. [36]**	0.17	0.31	0.44	1.03	5.22
**Proposed Algorithm**	0.18	0.34	0.49	1.13	5.77

**Table 9:** Hardware synthesis results of different architectures for realizing the modified hybrid median filter with 5×5 and 7×7 windows.

Xilinx Design Analyzer ${}^{1}$									
Device	xc7z045 - 2ffg900								
Design		5×5 mHMF				7×7 mHMF			
		BSN-Based		OMSN-Based		BSN-Based		OMSN-Based	
Slice Logic Utilization	Available	Used	Util.	Used	Util.	Used	Util.	Used	Util.
Slice Registers (#)	437,200	4916	1.12%	4,056	0.93%	11,139	2.55%	9344	2.14%
Slice LUTs (#)	218,600	4599	2.10%	3771	1.73%	9745	4.46%	8427	3.85%
Used as Memory (#)	70,400	74	0.11%	124	0.18%	104	0.15%	234	0.33%
RAM36E1 / FIFO36E1s	545	4	0.73%	4	0.73%	6	1.10%	6	1.10%
Minimum Period		2.800 ns		2.542 ns		2.803 ns		2.547 ns	
Maximum Frequency		357.143 MHz		393.391 MHz		356.761 MHz		392.619 MHz	

${}^{1}$ The EDA Tool was supported by the IC Design Education Center.

**Table 10:** Hardware synthesis result of the proposed visibility restoration algorithm.

Xilinx Design Analyzer			
Device	xc7z045 - 2ffg900		
Slice Logic Utilization	Available	Used	Utilization
Slice Registers (#)	437,200	57,848	13.23%
Slice LUTs (#)	218,600	53,569	24.51%
RAM36E1/FIFO36E1s	545	58	10.64%
RAM18E1/FIFO18E1s	1090	25	2.29%
Minimum Period	3.68 ns		
Maximum Frequency	271.67 MHz		

**Table 11:** Maximum processing speed for various video resolutions.

Video Resolution		Frame Size	Required Clock Cycles (#)	Processing Speed (fps)
Full HD (FHD)		1920×1080	2,076,601	130.8
Quad HD (QHD)		2560×1440	3,690,401	73.6
4K	UW4K	3840×1600	6,149,441	44.2
	UHD TV	3840×2160	8,300,401	32.7
	DCI 4K	4096×2160	8,853,617	30.7

**Table 12:** A comparative evaluation with other hardware designs.

Hardware Utilization	Park et al. [54]	Ngo et al. [18]	Proposed Design
Registers (#)	53,400	70,864	57,848
LUTs (#)	64,000	56,664	53,569
DSPs (#)	42	0	0
Memory (Mbits)	3.2	1.5	2.4
Maximum Processing Rate (Mpixel/s)	88.70	236.29	271.67
Maximum Attainable Video Resolution	SVGA	DCI 4K	DCI 4K

## References

[1] Chen X., Wang S., Shi C., Wu H., Zhao J., Fu J. (2019). Robust Ship Tracking via Multi-view Learning and Sparse Representation. J. Navig..

[2] Sengee N., Sengee A., Choi H.K. (2010). Image contrast enhancement using bi-histogram equalization with neighborhood metrics. IEEE Trans. Consum. Electron..

[3] Tan S.F., Isa N.A.M. (2019). Exposure Based Multi-Histogram Equalization Contrast Enhancement for Non-Uniform Illumination Images. IEEE Access.

[4] Ngo D., Kang B. (2018). Preprocessing for High Quality Real-time Imaging Systems by Low-light Stretch Algorithm. J. Inst. Korean. Electr. Electron. Eng..

[5] Ngo D., Lee S., Kang B. Nonlinear Unsharp Masking Algorithm. Proceedings of the 2020 International Conference on Electronics, Information, and Communication (ICEIC).

[6] Polesel A., Ramponi G., Mathews V. (2000). Image enhancement via adaptive unsharp masking. IEEE Trans. Image Process..

[7] Fries R., Modestino J. (1979). Image enhancement by stochastic homomorphic filtering. IEEE Trans. Signal Process..

[8] Kaufman H., Sid-Ahmed M. (1993). Hardware realization of a 2D IIR semisystolic filter with application to real-time homomorphic filtering. IEEE Trans. Circuits Syst. Video Technol..

[9] Lee Z., Shang S. (2016). Visibility: How Applicable is the Century-Old Koschmieder Model?. J. Atmos. Sci..

[10] Fattal R. (2008). Single image dehazing. ACM Trans. Graph..

[11] He K., Sun J., Tang X. (2011). Single Image Haze Removal Using Dark Channel Prior. IEEE Trans. Pattern Anal. Mach. Intell..

[12] Tarel J.P., Hautière N. Fast visibility restoration from a single color or gray level image. Proceedings of the 2009 IEEE 12th International Conference on Computer Vision.

[13] Zhu Q., Mai J., Shao L. (2015). A Fast Single Image Haze Removal Algorithm Using Color Attenuation Prior. IEEE Trans. Image Process..

[14] Berman D., Treibitz T., Avidan S. Non-local Image Dehazing. Proceedings of the 2016 IEEE Conference on Computer Vision and Pattern Recognition (CVPR).

[15] Ngo D., Lee S., Nguyen Q.H., Ngo T.M., Lee G.D., Kang B. (2020). Single Image Haze Removal from Image Enhancement Perspective for Real-Time Vision-Based Systems. Sensors.

[16] Papyan V., Elad M. (2016). Multi-Scale Patch-Based Image Restoration. IEEE Trans. Image Process..

[17] Park D., Park H., Han D.K., Ko H. Single image dehazing with image entropy and information fidelity. Proceedings of the 2014 IEEE International Conference on Image Processing (ICIP).

[18] Ngo D., Lee G.D., Kang B. (2019). A 4K-Capable FPGA Implementation of Single Image Haze Removal Using Hazy Particle Maps. Appl. Sci..

[19] Levin A., Lischinski D., Weiss Y. (2008). A Closed-Form Solution to Natural Image Matting. IEEE Trans. Pattern Anal. Mach. Intell..

[20] Li C., Zhang X. Underwater Image Restoration Based on Improved Background Light Estimation and Automatic White Balance. Proceedings of the 2018 11th International Congress on Image and Signal Processing, BioMedical Engineering and Informatics (CISP-BMEI).

[21] Lee S., Yun S., Nam J.H., Won C.S., Jung S.W. (2016). A review on dark channel prior based image dehazing algorithms. EURASIP J. Image Video Process..

[22] Zhu Y., Tang G., Zhang X., Jiang J., Tian Q. (2018). Haze removal method for natural restoration of images with sky. Neurocomputing.

[23] Park Y., Kim T.H. (2018). Fast Execution Schemes for Dark-Channel-Prior-Based Outdoor Video Dehazing. IEEE Access.

[24] Tufail Z., Khurshid K., Salman A., Fareed Nizami I., Khurshid K., Jeon B. (2018). Improved Dark Channel Prior for Image Defogging Using RGB and YCbCr Color Space. IEEE Access.

[25] He K., Sun J., Tang X. (2013). Guided Image Filtering. IEEE Trans. Pattern Anal. Mach. Intell..

[26] Gibson K.B., Vo D.T., Nguyen T.Q. (2012). An Investigation of Dehazing Effects on Image and Video Coding. IEEE Trans. Image Process..

[27] Kim G.J., Lee S., Kang B. (2018). Single Image Haze Removal Using Hazy Particle Maps. IEICE Trans. Fundam. Electron. Commun. Comput. Sci..

[28] Tang K., Yang J., Wang J. Investigating Haze-Relevant Features in a Learning Framework for Image Dehazing. Proceedings of the 2014 IEEE Conference on Computer Vision and Pattern Recognition.

[29] Ngo D., Lee S., Kang B. (2020). Robust Single-Image Haze Removal Using Optimal Transmission Map and Adaptive Atmospheric Light. Remote Sens..

[30] Choi L.K., You J., Bovik A.C. (2015). Referenceless Prediction of Perceptual Fog Density and Perceptual Image Defogging. IEEE Trans. Image Process..

[31] Cai B., Xu X., Jia K., Qing C., Tao D. (2016). DehazeNet: An End-to-End System for Single Image Haze Removal. IEEE Trans. Image Process..

[32] Li C., Guo J., Porikli F., Fu H., Pang Y. (2018). A Cascaded Convolutional Neural Network for Single Image Dehazing. IEEE Access.

[33] Golts A., Freedman D., Elad M. (2020). Unsupervised Single Image Dehazing Using Dark Channel Prior Loss. IEEE Trans. Image Process..

[34] Ren W., Pan J., Zhang H., Cao X., Yang M.H. (2020). Single Image Dehazing via Multi-scale Convolutional Neural Networks with Holistic Edges. Int. J. Comput. Vis..

[35] Li B., Ren W., Fu D., Tao D., Feng D., Zeng W., Wang Z. (2019). Benchmarking Single-Image Dehazing and Beyond. IEEE Trans. Image Process..

[36] Ngo D., Lee G.D., Kang B. (2019). Improved Color Attenuation Prior for Single-Image Haze Removal. Appl. Sci..

[37] Ngo D., Kang B. A New Data Preparation Methodology in Machine Learning-based Haze Removal Algorithms. Proceedings of the 2019 International Conference on Electronics, Information, and Communication (ICEIC).

[38] Ngo D., Kang B. (2018). Improving Performance of Machine Learning-based Haze Removal Algorithms with Enhanced Training Database. J. Inst. Korean Electr. Electron. Eng..

[39] Cho H., Kim G.J., Jang K., Lee S., Kang B. (2015). Color Image Enhancement Based on Adaptive Nonlinear Curves of Luminance Features. J. Semicond. Technol. Sci..

[40] Tarel J.P., Hautiere N., Caraffa L., Cord A., Halmaoui H., Gruyer D. (2012). Vision Enhancement in Homogeneous and Heterogeneous Fog. IEEE Intell. Transp. Syst. Mag..

[41] Ancuti C., Ancuti C.O., De Vleeschouwer C. D-HAZY: A dataset to evaluate quantitatively dehazing algorithms. Proceedings of the 2016 IEEE International Conference on Image Processing (ICIP).

[42] Ma K., Liu W., Wang Z. Perceptual evaluation of single image dehazing algorithms. Proceedings of the 2015 IEEE International Conference on Image Processing (ICIP).

[43] Ancuti C.O., Ancuti C., Timofte R., De Vleeschouwer C. O-HAZE: A Dehazing Benchmark with Real Hazy and Haze-Free Outdoor Images. Proceedings of the 2018 IEEE/CVF Conference on Computer Vision and Pattern Recognition Workshops (CVPRW).

[44] Ancuti C.O., Ancuti C., Timofte R., De Vleeschouwer C. (2018). I-HAZE: A dehazing benchmark with real hazy and haze-free indoor images. arXiv.

[45] Wang Z., Bovik A., Sheikh H., Simoncelli E. (2004). Image quality assessment: from error visibility to structural similarity. IEEE Trans. Image Process..

[46] Yeganeh H., Wang Z. (2013). Objective Quality Assessment of Tone-Mapped Images. IEEE Trans. Image Process..

[47] Zhang L., Zhang L., Mou X., Zhang D. (2011). FSIM: A Feature Similarity Index for Image Quality Assessment. IEEE Trans. Image Process..

[48] Hautiere N., Tarel J.P., Aubert D., Dumont E. (2008). Blind Contrast Enhancement Assessment by Gradient Ratioing at Visible Edges. Image Anal. Stereol..

[49] Jack K., Jack K. (2005). Chapter 9 - NTSC and PAL Digital Encoding and Decoding. Video Demystified.

[50] STD90 Samsung 0.35μm 3.3V CMOS Standard Cell Library for Pure Logic/MDL Products. https://www.chipfind.net/datasheet/samsung/std90.htm.

[51] Knuth D.E. (1998). The Art of Computer Programming, Volume 3: Sorting and Searching.

[52] Zynq-7000 SoC Data Sheet: Overview (DS190). https://www.xilinx.com/support/documentation/data_sheets/ds190-Zynq-7000-Overview.pdf.

[53] (2006). IEEE Standard for Verilog Hardware Description Language. IEEE Std 1364-2005.

[54] Park Y., Kim T.H. A video dehazing system based on fast airlight estimation. Proceedings of the 2017 IEEE Global Conference on Signal and Information Processing (GlobalSIP).

